# Descending pain modulation in irritable bowel syndrome (IBS): a systematic review and meta-analysis

**DOI:** 10.1186/s13643-015-0162-8

**Published:** 2015-12-10

**Authors:** Rosemary J. Chakiath, Philip J. Siddall, John E. Kellow, Julia M. Hush, Mike P. Jones, Anna Marcuzzi, Paul J. Wrigley

**Affiliations:** Sydney Medical School Northern, University of Sydney, Sydney, NSW Australia; Pain Management Research Institute, Kolling Institute, Northern Sydney Local Health District, St Leonards, Sydney, NSW Australia; Department of Pain Management, HammondCare, Greenwich Hospital, Sydney, NSW Australia; Kolling Institute of Medical Research, Royal North Shore Hospital, St Leonards, Sydney, NSW Australia; Department of Gastroenterology, Royal North Shore Hospital, St Leonards, Sydney, NSW Australia; Discipline of Physiotherapy, Department of Health Professions, Faculty of Medicine and Health Sciences, Macquarie University, North Ryde, Sydney, NSW Australia; The Centre for Physical Health, Macquarie University, North Ryde, Australia; Psychology Department, Macquarie University, North Ryde, Sydney, NSW Australia

**Keywords:** Chronic pain, Irritable bowel syndrome, Central pain mechanisms, Central pain modulators, Descending pain modulation

## Abstract

**Background:**

Irritable bowel syndrome (IBS) is a common functional gastrointestinal disorder. While abdominal pain is a dominant symptom of IBS, many sufferers also report widespread hypersensitivity and present with other chronic pain conditions. The presence of widespread hypersensitivity and extra-intestinal pain conditions suggests central nervous dysfunction. While central nervous system dysfunction may involve the spinal cord (central sensitisation) and brain, this review will focus on one brain mechanism, descending pain modulation.

**Method/design:**

We will conduct a comprehensive search for the articles indexed in the databases Ovid MEDLINE, Ovid Embase, Ovid PsycINFO and Cochrane Central Register of Controlled Trial (CENTRAL) from their inception to August 2015, that report on any aspect of descending pain modulation in irritable bowel syndrome. Two independent reviewers will screen studies for eligibility, assess risk of bias and extract relevant data. Results will be tabulated and, if possible, a meta-analysis will be carried out.

**Discussion:**

The systematic review outlined in this protocol aims to summarise current knowledge regarding descending pain modulation in IBS.

**Systematic review registration:**

PROSPERO CRD42015024284

## Background

Irritable bowel syndrome (IBS) is a chronic gastrointestinal disorder characterised by abdominal pain, discomfort and altered bowel habit [1].

A significant amount of research suggests that the abdominal pain associated with IBS is due to peripheral changes occurring in the gut (visceral nociceptive hypersensitivity) [2–4]. However, not all people with IBS have evidence of visceral hypersensitivity, and some exhibit nociceptive hypersensitivity in non-visceral structures (e.g. cutaneous structures) [5, 6]. Such non-visceral hypersensitivity may even be widespread extending to regions quite distant from the site of concern. In addition, other non-visceral, extra-intestinal chronic pain conditions frequently occur with IBS such as fibromyalgia, chronic low back pain, chronic fatigue, dysmenorrhea and cystitis (bacterial and interstitial cystitis) [7].

While peripheral mechanisms remain important in IBS, when widespread hypersensitivity and extra-intestinal pain syndromes occur, this suggests a central nervous system involvement [8–11]. Central nervous system nociceptive processing is commonly divided into spinal cord processes (central sensitisation) and those occurring in the brain (e.g. descending pain modulation) [12].

The term descending pain modulation (DPM) is used to describe the process mediated by pathways descending from the brain stem that modulate spinal cord ascending nociceptive transmission. Descending neural modulatory circuits from the brain are able to inhibit or facilitate ascending nociceptive transmission and are also known to be influenced by cognitive processes and mood. Ultimately, changes in DPM are felt to profoundly influence pain perception.

We will undertake a systematic review of the research examining descending pain modulation in IBS. As part of this review, we will examine the influence of cognitive processes and mood on descending modulation [13].

This systematic review will summarise current knowledge and identify unresolved questions regarding descending pain modulation in IBS.

### Research questions

This systematic review aims to answer the following research questions: (1) Do people with IBS when compared with healthy controls demonstrate a consistent difference in descending pain modulation as assessed by neurophysiological testing (change in pain intensity and waveform analysis) and (2) If descending pain modulation is altered in people with IBS compared with healthy controls, what is the magnitude of that difference?

## Methods and design

### Study registration

The protocol of this systematic review has been registered on PROSPERO 2015 [14] (registration number: CRD42015024284). The protocol has been conducted and reported using the Preferred Reporting Items for Systematic Reviews and Meta-Analyses (PRISMA) statement guidelines [15].

### Search strategy for identification of relevant studies

To identify the relevant literature, electronic searches will be conducted in the following databases: Ovid MEDLINE, Ovid Embase, Ovid PsycINFO and Cochrane Central Register of Controlled Trial (CENTRAL) from their inception to August 2015. A comprehensive search strategy has been designed with the assistance of an experienced research librarian and adjusted to account for differences in indexing across databases. Articles identified through reference lists of included studies and relevant systematic reviews will be considered for inclusion based on their title. In addition, cross-checking of references, citations in review papers, and communication with scientists who have been working in the field will also be carried out. Non-English language studies will be included, where a translation can be made available. See Appendix for the MEDLINE search strategy.

### Eligibility criteria

#### Participants

We will include studies of adults (18 years or older) who have been diagnosed with IBS where the diagnosis has been made according to the ROME criteria [16].

Studies of adults with comorbid intestinal disorders (e.g. gastroesophageal reflux dysphagia, esophageal spasms and functional dyspepsia) and/or extra-intestinal disorders (e.g. low back pain, fibromyalgia, chronic fatigue, dysmenorrhea and both bacterial and interstitial cystitis) alongside IBS will also be included.

#### Outcome measure

The outcome of interest is descending pain modulation function in people with IBS as assessed by neurophysiological testing i.e. conditioned pain modulation (CPM) or offset analgesia (OA). The neurophysiological protocol has to use a painful test stimulus assessed both prior to and during and/or after the presentation of a conditioning stimulus. Descending pain modulation in the control group would be expected to be quantified as normally responsive. Those studies where descending pain modulation in control participants are not quantified as normally responsive, will be examined as a potential source of variability and will be considered as a candidate for a stratified meta-analysis.

#### Types of studies

The study designs considered relevant to this review include cross-sectional studies, case-control studies, cohort studies and randomised controlled trials. Qualitative studies will be excluded. We will exclude intervention studies if assessment of descending pain modulation is only reported after treatment (e.g. drug administration, surgical techniques). Reference lists of relevant systematic reviews will be checked in order to identify relevant primary studies, but systematic reviews will otherwise be excluded.

#### Screening of studies

Following the initial database search, duplicate papers will be removed and two reviewers will independently screen titles and abstracts of selected studies to identify those, which are potentially suitable and meet the inclusion criteria.

These studies will then be reviewed in full text by two reviewers to determine their eligibility using a standardised eligibility criteria sheet. Reasons for exclusion of papers will be recorded when screening full papers. Any discrepancies in this final list will be discussed between the reviewers, with an independent reviewer consulted if a consensus cannot be attained.

### Data extraction

Data from the included studies will be extracted using a standardised eligibility criteria sheet. Any discrepancies in this final list will be discussed between the reviewers, with an independent reviewer consulted if a consensus cannot be attained. In addition, authors of papers will be contacted where there is incomplete data or data that needs further clarification.

The following data will be collected from the included studies. General study information: authors, year of publication, sample size, language; study design: cross-sectional, case-control, observational study or clinical trial; clinical setting: primary care, specialist clinic, hospital outpatient department; participant characteristics: age, gender, classification or diagnostic criteria used, duration of pain, severity of pain; measure of descending pain modulation: conditioning stimulus, test stimulus, outcome measure and time points outcome measure were recorded; measure of cognitive processes and mood: assessment type used.

### Risk of bias assessment

Several risks of bias tools including the non-randomised studies of interventions (ACROBAT-NRSI) [17] and the quality assessment tool for diagnostic accuracy studies-2 (QUADAS-2) [18] were evaluated according to the guidelines outlined in the Cochrane handbook for systematic reviews. The domains of bias listed in these tools were developed for randomised or non-randomised clinical trials and were not suitable for the current review.

The Quality in Prognostic Studies (QUIPS) tool developed by Hayden, van der Windt, Cartwright, Cote´, Bombardier [19] was also considered for risk of bias assessment. The QUIPS tool is comprised of six important domains of bias and includes prompting items to help inform the judgement of risk of bias. Each bias domain is then individually rated as having a high, moderate or low risk of bias. However, not all items were considered relevant to this review. For example, while attrition is important in longitudinal studies, the studies central to the current review are cross-sectional in nature. In the event that longitudinal studies are included, only baseline responses will be evaluated. The QUIPS tool was considered the closest in design to that required for this review. However, the use of a graded risk of bias assessment within domains and an overall quantitative risk score was considered problematic. It was felt that ranking risk criteria within each category as low, medium or high introduced further subjectivity to the assessment.

With these limitations taken together, a modified version of the QUIPS risk of bias tool was employed. The risk of bias tool developed for the current review consists of six categories including: (1) The criteria outline by the ROME foundation [16] to assess whether cases are representative of population, (2) comparability of cases and controls, (3) participants clearly defined, (4) quality of CPM protocol, (5) blinding and (6) statistical methods and study size. As used in the Cochrane risk of bias tool for non-randomised studies of interventions (ACROBAT-NRSI) [17], each study will be rating as having met (yes) or not met (no) each bias domain according to the guidelines outlined in the tool (see Table 1). In studies where there is uncertainty in a category, the category will be rated as “unclear”. A summary of the results for all included studies will be provided and a qualitative analysis will be undertaken. Inter-rater agreement of the risk of bias tool will be tested by calculating a kappa statistic. Any disagreements will be discussed between the reviewers, with an independent reviewer consulted if consensus cannot be attained.

**Table 1:** Risk of bias tool

Author and year of publication		
Study identifier		
Reviewer		
Bias domain	Issues to consider for judging overall rating of “risk of bias”	Rating
		Yes/no/unclear
1. Cases representative of population		
Diagnosis of IBS	Diagnosis of IBS made according to the ROME criteria	
2. Comparability of cases and controls		
Inclusion/exclusion criteria for healthy controls specified	Ensure controls also use ROME criteria	
	Ensure controls are comparable to cases in terms of age and gender	
3. Participants clearly defined		
All clinical and demographical characteristics described	• Clinical and demographical characteristics were fully described (yes/no)	
	• Sample size (yes/no)	
	• Mean age (yes/no)	
	• Gender percentages (yes/no)	
	• Mean pain duration (yes/no)	
	• Mean pain intensity (yes/no)	
Clinical setting	Clinical setting clearly specified	
4. Quality of conditioned pain modulation (CPM) protocol		
Testing explicitly described	• Testing protocol (CPM) explicitly described (yes/no)	
	• Number of examiners preforming assessments (yes/no)	
	• Equipment used (yes/no)	
	• Clear description of procedure including number of assessments and sites (yes/no)	
5. Blinding		
Assessor blinding	Assessors were blinded to participant group or condition	
6. Statistical methods and study size		
Method of determining study size described and appropriate	Method of determining study design was explicitly described and appropriate	
Confounding variables controlled	Were confounding variables controlled? (e.g. through exclusion criteria, matched controls, statistical analysis)	
	• Medication (yes/no)	
	• Caffeine (yes/no)	
	• Day of testing (yes/no)	
	• Pain on test day (yes/no)	
	• Time of day testing was undertaken (yes/no)	
	• Menstrual cycle phase (yes/no)	
	• Other pain or sensory conditions (yes/no)	
Presentation of analytical strategy	Statistical analysis explicitly described and appropriate	

### Data analysis

Results for descending pain modulation function within a study will be tabulated according to the type of measure used for assessment (e.g. change in pain intensity rating or neurophysiological measure). The effect size for descending pain modulation function within a study will then be calculated using means and standard deviations at baseline (prior to testing) and during or following testing in the patient and control populations. When a variety of effect size measures are reported, they will be converted to a common measure when that is possible, such as to Cohen’s d [20] or correlation values [21].

For each measure available, the random effects model of DerSimonian and Laird [22] will be used to create pooled effect sizes which will be reported along with 95 % confidence intervals. When there is no or little between-study variability, the random-effects model is mathematically equivalent to the fixed-effects model. Initially, all studies reporting each measure will be included. Homogeneity of effect sizes will be assessed via inspection of forest plots and via Cochrane’s test [22] and reported with I^2^ values [23] to quantify the degree of between-study variability. The potential for publication bias will be assessed through inspection of funnel plots and via Egger’s test [24]. Where substantial or statistically significant between-study variability is detected and the candidate sources of such variability can be identified, stratified meta-analyses will be conducted.

In addition, if there are adequate data in the included studies, a separate analysis will also be carried out to investigate the influence that cognitive processes (e.g. hypervigilance, catastrophising and self-efficacy) and mood (e.g. depression and anxiety) have on neurophysiological measures of descending modulation.

Lastly, if the heterogeneity of the data does not allow pooling and quantitative statistics, a narrative synthesis of the findings will be undertaken and the implications critically discussed.

## Discussion

This systematic review will consolidate our current knowledge of descending pain modulation in IBS.

## Appendix

### Ovid MEDLINE search strategy

1. irritable Bowel Syndrome/
2. (visceral adj pain).mp. [mp = title, abstract, original title, name of substance word, subject heading word, keyword heading word, protocol supplementary concept word, rare disease supplementary concept word, unique identifier]
3. (visceral adj hypersensitivity).mp.
4. abdominal pain/
5. or/1–4
6. pain perception/
7. nociception/
8. pain threshold/
9. sensory profile*.mp.
10. pain, referred/
11. sensory hypersensitivity.mp.
12. hypersensitive*.mp.
13. hyp*algesia.mp.
14. hyp*esthesia.mp.
15. dys*esthesia.mp.
16. par*esthesia.mp.
17. hyperpathia.mp.
18. allodynia.mp.
19. analgesia.mp.
20. central pain.mp.
21. exp somatosensory disorders/
22. somatosensory.mp.
23. central sensit*.mp.
24. peripheral sensit*.mp.
25. somatic hypersensitivity.mp.
26. cutaneous hyp*sensitivity.mp.
27. spinal sensitisation.mp.
28. psychophysic*.mp.
29. quantitative sensory test*.mp.
30. QST.mp.
31. detection threshold.mp.
32. ((cold or warm) adj detection).mp.
33. electrophysiologic*.mp
34. experim* adj pain.mp.
35. pain adj measurement.mp.
36. pain adj test.mp.
37. temporal summation.mp.
38. pain adj threshold.mp.
39. wind up.mp.
40. pain adj tolerance.mp.
41. (pain adj processing).mp.
42. spinal reflex*.mp.
43. reflex receptive field or RRF.mp.
44. nociceptive withdrawal reflex.mp.
45. nociceptive flexion reflex.mp.
46. NFR.mp.
47. NWR.mp.
48. (RIII adj reflex).mp.
49. conditioned pain modulation or CPM.mp.
50. cold pressor test.mp.
51. diffuse noxious inhibitory control/
52. DNIC.mp.
53. offset adj analgesia.mp.
54. neural inhibition.mp.
55. descending pain modulation.mp.
56. endogenous pain modulation.mp.
57. central pain modulation.mp.
58. or/6–57
59. 5 and 58
60. qualitative research/
61. 59 not 60
62. limit 61 to humans

## Abbreviations

CPM: conditioned pain modulation
DPM: descending pain modulation
IBS: irritable bowel syndrome
OA: offset analgesia
QUADAS-2: quality assessment tool for diagnostic accuracy studies-2
QUIPS: quality in prognostic studies
